# In Vitro and In Vivo Efficacy of the Essential Oil from the Leaves of *Annona amazonica* R.E. Fries (Annonaceae) Against Liver Cancer

**DOI:** 10.3390/molecules30153248

**Published:** 2025-08-02

**Authors:** Maria V. L. de Castro, Milena C. F. de Lima, Gabriela A. da C. Barbosa, Sabrine G. Carvalho, Amanda M. R. M. Coelho, Luciano de S. Santos, Valdenizia R. Silva, Rosane B. Dias, Milena B. P. Soares, Emmanoel V. Costa, Daniel P. Bezerra

**Affiliations:** 1 Gonçalo Moniz Institute, Oswaldo Cruz Foundation (IGM-FIOCRUZ/BA), Salvador 40296-710, BA, Brazil; mvictorialimac@gmail.com (M.V.L.d.C.); gabibarbosa.alves@gmail.com (G.A.d.C.B.); sabrinegama17@gmail.com (S.G.C.); amandaribeirocoelho@hotmail.com (A.M.R.M.C.); luciano.biomed@gmail.com (L.d.S.S.); valdeniziar@gmail.com (V.R.S.); rosanebd@gmail.com (R.B.D.); milena.soares@fiocruz.br (M.B.P.S.); 2 Department of Chemistry, Institute of Exact Sciences, Federal University of Amazonas (UFAM), Manaus 69080-900, AM, Brazil; mcampelo@ufam.edu.br; 3 Department of Biological Sciences, State University of Feira de Santana, Feira de Santana 44036-900, BA, Brazil; 4 SENAI Institute for Innovation in Advanced Health Systems, SENAI CIMATEC, Salvador 41650-010, BA, Brazil; 5 Postgraduate Program in Chemistry, Institute of Exact Sciences, Federal University of Amazonas (UFAM), Manaus 69080-900, AM, Brazil

**Keywords:** *Annona amazonica*, essential oil, liver cancer, antitumor

## Abstract

*Annona amazonica* R.E. Fries (synonyms *Annona amazonica* var. *lancifolia* R.E. Fries), popularly known in Brazil as “envireira”, is a tropical tree belonging to the Annonaceae family and is traditionally used as a food source. In this work, the in vitro and in vivo anti-liver cancer effects of essential oil (EO) from *A. amazonica* leaves were investigated for the first time. The chemical composition of the EO was evaluated via GC–MS and GC–FID. The alamar blue assay was used to evaluate the cytotoxicity of EOs against different cancerous and noncancerous cell lines. Cell cycle analyses, YO-PRO-1/PI staining, and rhodamine 123 staining were performed via flow cytometry in HepG2 cells treated with EO. The in vivo antitumor activity of EO was evaluated in NSG mice that were xenografted with HepG2 cells and treated with EO at a dose of 60 mg/kg. The major constituents (>5%) of the EO were (*E*)-caryophyllene (32.01%), 1,8-cineole (13.93%), α-copaene (7.77%), α-humulene (7.15%), and α-pinene (5.13%). EO increased apoptosis and proportionally decreased the number of viable HepG2 cells. The induction of DNA fragmentation and cell shrinkage together with a significant reduction in the ΔΨm in EO-treated HepG2 cells confirmed that EO can induce apoptosis. A significant 39.2% inhibition of tumor growth in vivo was detected in EO-treated animals. These data indicate the anti-liver cancer potential of EO from *A. amazonica* leaves.

## 1. Introduction

Liver cancer is a global health issue, with hepatocellular carcinoma being the most common type of primary liver cancer. It is the sixth most diagnosed cancer, with an estimated 865,269 new cases, and the third leading cause of cancer-related deaths, accounting for approximately 757,948 deaths worldwide in 2022 [1]. In advanced stages of the disease, systemic therapy is commonly employed. This includes combinations of tyrosine kinase inhibitors (e.g., sorafenib, regorafenib, lenvatinib, and cabozantinib) and immune checkpoint inhibitors, such as anti-PD-1 agents (e.g., nivolumab and pembrolizumab), anti-PD-L1 agents (e.g., atezolizumab), and/or anti-CTLA-4 agents (e.g., ipilimumab) [2,3,4].

In contrast to recent advances in liver cancer treatment, the American Cancer Society reported that from 2014 to 2020, the 5-year relative survival rates for patients with liver cancer were 37% for those with localized tumors, 13% for those with regional tumors, and 3% for those with distant tumors, resulting in an overall survival rate of only 22% [5]. These data reinforce the need to explore new therapeutic options for the treatment of liver cancer.

Essential oils (EOs) are complex mixtures of volatile molecules isolated from aromatic plants that exhibit promising cytotoxic effects against various types of cancer, including breast, liver, colon, and lung cancer cell lines, through diverse mechanisms of action [6,7,8,9,10]. Owing to their natural origin and multitarget effects, EOs are promising candidates for complementary cancer treatments.

*Annona amazonica* R.E. Fries (synonyms *Annona amazonica* var. *lancifolia* R.E. Fries), popularly known in Brazil as “envireira”, is a tropical tree belonging to the Annonaceae family. It is a tree that is 20 to 25 m tall and is distinguished from other species by having very small flowers, with congenital outer and inner petals and glaucous fruits. This species is native to a region stretching from Costa Rica to northern Brazil, where it is traditionally used as a food source [11,12,13].

Previous phytochemical investigations of *A. amazonica* have reported the presence of cyanogenic compounds in its leaves, seeds, and fruits [14]. Pinheiro et al. [15] successfully isolated acanthoic acid in high yield from the stem of *A. amazonica* and demonstrated its trypanocidal activity. More recently, Alcantara et al. [16] identified (*E*)-caryophyllene and linalool as the major constituents of EOs extracted from the leaves and branches of *A. amazonica*, respectively. However, the antitumor potential of this species remains unexplored.

This work aimed to determine the chemical composition of *A. amazonica* leaf EO and investigate its anticancer potential. In the present study, the in vitro and in vivo anti-liver cancer effects of the EO derived from *A. amazonica* leaves were reported for the first time.

## 2. Results and Discussion

### 2.1. Chemical Constituents of the EO from the Leaves of A. amazonica

The EO obtained from the dried leaves of *A. amazonica* by hydrodistillation exhibited a light green color, a pronounced aroma, and an average yield of 0.18 ± 0.02% relative to the dry weight of the analyzed samples. Figure S1 illustrates the experimental workflow performed. The chemical composition of the oil was characterized via gas chromatography–mass spectrometry (GC–MS) and gas chromatography–flame ionization detection (GC–FID) (Figure S2). The identification of the chemical constituents was based on comparisons of the obtained mass spectra (Figures S3–S7), retention indices, and previously reported data.

**Figure 1:**
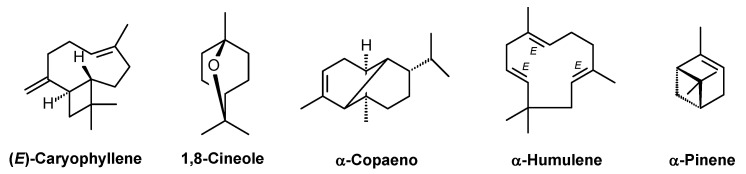
Principal constituents identified in the EO hydrodistillation from the leaves of *A. amazonica*.

As a result of the qualitative and quantitative analyses, 49 compounds were identified, representing 98.17% of the total EO composition. All the detected constituents belonged to the terpenoid class, with a predominance of sesquiterpenes, which accounted for 68.50% of the total composition, whereas monoterpenes constituted 29.67% of the total composition. The major constituents (>5%) of the EO were (*E*)-caryophyllene (32.01%), 1,8-cineole (13.93%), α-copaene (7.77%), α-humulene (7.15%), and α-pinene (5.13%) (Table 1 and Figure 1).

These findings are consistent with those reported by Alcantara et al. [16], who identified (*E*)-caryophyllene as the major constituent of the EO extracted from the leaves of *A. amazonica*. In other *Annona* species, (*E*)-caryophyllene has also been identified among the principal components of the EOs from *A. cherimola*, *A. foetida*, *A. emarginata*, *A. glabra*, atemoya/Abdel Razek (hybrid of *A. squamosa* and *A. cherimola*), *A. reticulata*, *A. squamosa*, *A. leptopetala*, *A. neoinsignis*, *A. pickelii*, and *A. salzmannii* [19,20,21,22,23,24].

In addition, α-copaene, α-humulene, and/or α-pinene have also been previously reported as constituents of EOs from other *Annona* species [19,20,21,22,23,24,25,26,27]. These results suggest that the EO of *A. amazonica* shares similar chemical constituents with those of other *Annona* species. Nonetheless, variations in EO composition are common and may be influenced by various factors, including soil characteristics, climatic conditions, and plant age [28].

### 2.2. In Vitro Cytotoxic Effects of EO from the Leaves of A. amazonica

The in vitro cytotoxic activities of the EO from *A. amazonica* leaves were investigated via the alamar blue assay after 72 h of incubation. Table 2 displays the half-maximal inhibitory concentration (IC_50_) values found. EO had IC_50_ values ranging from 14.72 to 44.07 μg/mL for the human liver cancer cell line HepG2 and the human breast cancer cell line MCF-7. Doxorubicin was used as a positive control and had IC_50_ values ranging from 0.04 to 0.62 μg/mL for the human liver cancer cell line HepG2 and the human breast cancer cell line MDA-MB-231. For the noncancerous cell line MRC-5, EO had an IC_50_ value of 39.41 μg/mL, whereas doxorubicin had an IC_50_ value of 0.43 μg/mL.

The cytotoxic properties of EOs from *Annona* species have been previously reported and include those of EOs derived from *A. cherimola*, *A. glabra*, Atemoya/Abdel Razek, *A. squamosa*, *A. leptopetala*, *A. senegalensis*, *A. sylvatica*, *A. pickelii*, *A. salzmannii*, *A. vepretorum*, *A. neoinsignis*, and *A. muricata* [20,21,23,24,25,26,27,29,30,31]. Like the EO from *A. amazonica* leaves, (*E*)-caryophyllene is among the major constituents of the oils extracted from *A. cherimola*, *A. glabra*, Atemoya/Abdel Razek, *A. squamosa*, *A. leptopetala*, *A. pickelii*, *A. neoinsignis*, and *A. salzmannii* [20,21,23,24].

In contrast, the cytotoxic activity of the EO extracted from *A. senegalensis* leaves was related to the presence of caryophyllene oxide, as reported by Ahmed et al. [29]. Furthermore, spathulenol was identified as one of those responsible for the cytotoxicity observed in EO obtained from *A. vepretorum* leaves [25]. Moreover, the mixture of minor and major constituents of each EO may contribute to its pharmacological activity.

### 2.3. Apoptosis Induction by A. amazonica Leaf EO

In a new set of experiments, the induction of apoptosis by EO from *A. amazonica* leaves was evaluated via different flow cytometry assays at concentrations of 10, 5, and 2.5 μg/mL after 24 and 48 h of incubation with the liver cancer cell line HepG2, which is the cell line most sensitive to EO cytotoxicity.

**Figure 2:**
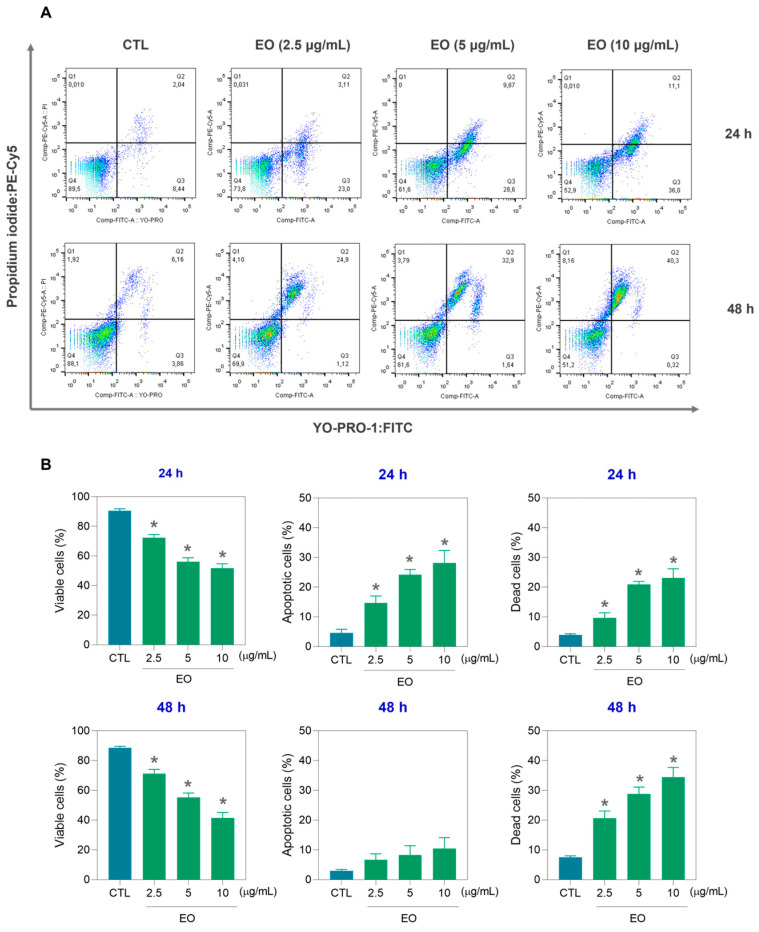
Apoptotic response of HepG2 cells to *A. amazonica* leaf EO after 24 and 48 h of treatment. (**A**) Representative flow cytometry dot plots showing the distribution of viable, apoptotic, and dead cells. (**B**) Quantitative analysis of the following cell populations: viable cells (YO-PRO-1^−^/PI^−^), apoptotic cells (YO-PRO-1^+^/PI^−^), and dead cells (YO-PRO-1^+^/PI^+^ plus YO-PRO-1^−^/PI^+^). The cells treated with 0.2% DMSO served as the vehicle control (CTL). The results are expressed as the mean ± SEM of three independent experiments performed in triplicate. * *p* < 0.05 vs. the control group (CTL), as determined by one-way ANOVA followed by Dunnett’s post hoc test.

First, the viability of HepG2 cells was assessed by staining with YO-PRO-1 together with propidium iodide (PI) (Figure 2). YO-PRO-1 staining is used as an early marker of apoptosis, whereas PI staining is used as a marker of cell membrane integrity [32]. In this assay, YO-PRO-1^−^/PI^−^ were considered viable; YO-PRO-1^+^/PI^−^ were considered apoptotic cells; and YO-PRO-1^+^/PI^+^ plus YO-PRO-1^−^/PI^+^ were considered dead cells, without identification of the type of cell death. Interestingly, EO increased apoptosis after 24 h of incubation, whereas dead cells predominated after 48 h of incubation. The number of viable cells decreased proportionally.

**Figure 3:**
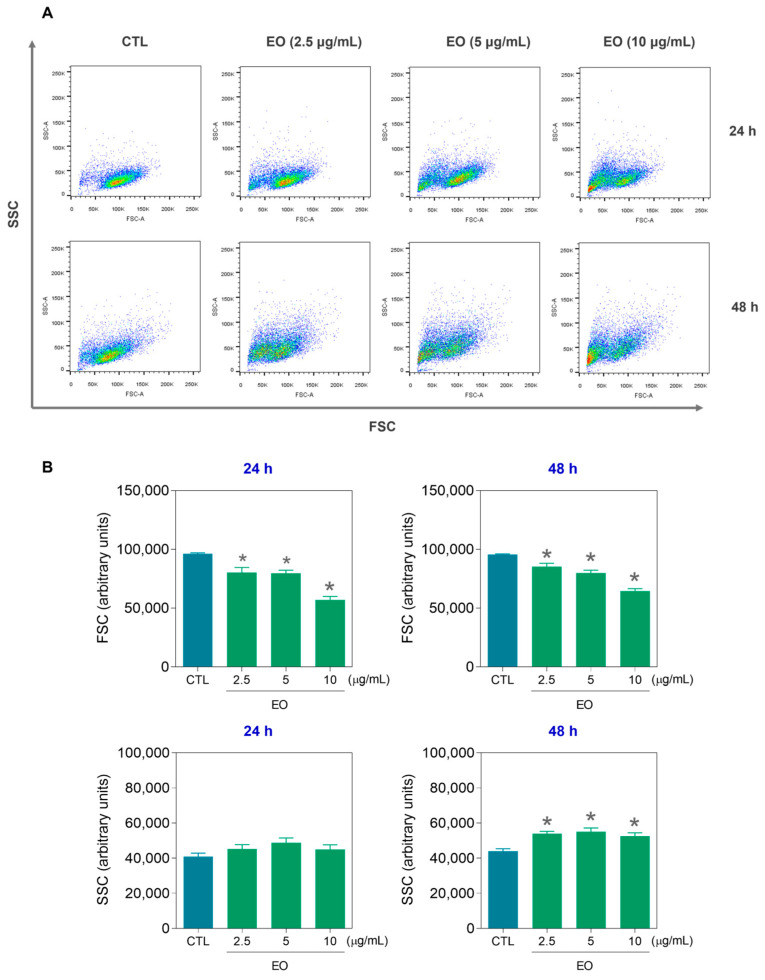
Effects of *A. amazonica* leaf EO on the size and internal complexity of HepG2 cells, as assessed by flow cytometry at 24 and 48 h after treatment. (**A**) Representative dot plots showing forward scatter (FSC) and side scatter (SSC) profiles, which reflect cell size and granularity, respectively. (**B**) Quantitative analysis of FSC and SSC values. The cells treated with 0.2% DMSO served as the vehicle control (CTL). The data are expressed as the means ± SEMs of three independent experiments performed in triplicate. * *p* < 0.05 vs. CTL, as determined by one-way ANOVA followed by Dunnett’s post hoc test.

**Figure 4:**
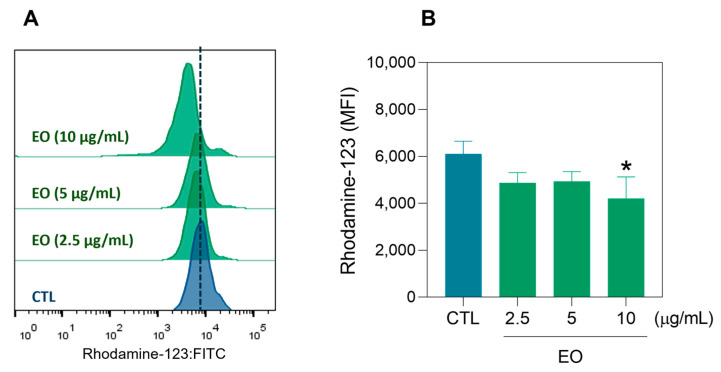
The ΔΨm in HepG2 cells after 24 h of treatment with *A. amazonica* leaf EO assessed via rhodamine-123 staining and flow cytometry. (**A**) Representative histograms obtained by flow cytometry. (**B**) Quantitative analysis of ΔΨm. The cells treated with 0.2% DMSO served as the vehicle control (CTL). The results are expressed as the mean fluorescence intensity (MFI) and represent the mean ± SEM of three independent experiments conducted in triplicate. * *p* < 0.05 vs. CTL, as determined by one-way ANOVA followed by Dunnett’s post hoc test.

The size and internal complexity of the HepG2 cells were also assessed via flow cytometry at 24 and 48 h after EO treatment (Figure 3). Forward scatter (FSC) was used as a cell size parameter and side scatter (SSC) was used as a cell granularity parameter. The reduction in FSC after 24 and 48 h of incubation and the increase in SSC after 48 h of incubation indicated cell shrinkage and nuclear condensation, characteristics of apoptotic cell death. Furthermore, the mitochondrial membrane potential (ΔΨm) was quantified in HepG2 cells via rhodamine-123 staining after 24 h of incubation with EO (Figure 4). A significant reduction in the ΔΨm was detected at a concentration of 10 μg/mL, confirming that the EO can induce apoptosis.

**Figure 5:**
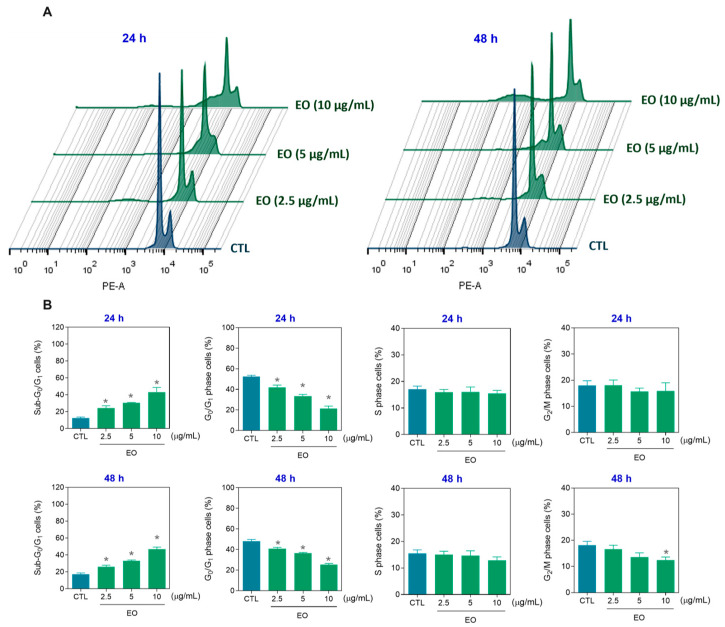
Cell cycle distribution of HepG2 cells after treatment with *A. amazonica* leaf EO for 24 and 48 h. (**A**) Representative histograms obtained by flow cytometry. (**B**) Quantitative analysis of the percentage of cells in the sub-G_0_/G_1_ (cells with fragmented DNA), G_0_/G_1_, S, and G_2_/M fractions. The cells treated with 0.2% DMSO served as the vehicle control (CTL). The data are expressed as the means ± SEMs of three independent experiments performed in triplicate. * *p* < 0.05 vs. CTL, as determined by one-way ANOVA followed by Dunnett’s post hoc test.

Next, the cell cycle and internucleosomal DNA fragmentation were detected in EO-treated HepG2 cells. For this purpose, PI staining was used to determine the cellular DNA content, and the G_0_/G_1_ (2n), S (2–4n), and G_2_/M (4n) cell phases were quantified (Figure 5). All cells with a DNA content less than 2n (sub-G_0_/G_1_ cells) were considered to have fragmented DNA. At concentrations of 10, 5, and 2.5 μg/mL, EO increased the percentage of HepG2 cells with fragmented DNA by 43.3, 30.3, and 24.3% after 24 h (vs. 12.6% of the control) and by 46.8, 33.1, and 25.9% after 48 h (vs. 17.4% of the control), respectively. The cell cycle phases (G_0_/G_1_, S, and G_2_/M) were proportionally reduced in EO-treated HepG2 cells. These data are also in agreement with cell death caused by apoptosis.

Previous studies revealed that EO extracted from *A. squamosa* pericarp induced apoptosis in SMMC-7721 hepatocellular carcinoma cells, as reported by Chen et al. [31], whereas the EO obtained from *A. neoinsignis* leaves caused apoptosis in HepG2 cells, as reported by Souza et al. [24]. Similarly, EO obtained from *A. vepretorum* leaves promoted apoptosis in B16-F10 melanoma cells [25]. In this study, we demonstrated for the first time that the EO extracted from *A. amazonica* causes apoptotic cell death in HepG2 cells.

(*E*)-Caryophyllene, the main constituent of the EO of *A. amazonica* leaves, has been previously reported as a potential antitumor agent [33]. It induces apoptosis and decreases angiogenesis in colorectal cancer cells [34]. This compound also causes S-phase cell cycle arrest and death in ovarian cancer cells [35] and reduces STAT-3/mTOR/AKT signaling in bladder cancer cells [36]. (*E*)-Caryophyllene also causes G_1_ cell cycle arrest, reduces sphingosine kinase 1, and potentiates the effects of cisplatin in lung cancer cells [37,38,39].

### 2.4. In Vivo Effects of A. amazonica Leaf EO on Liver Cancer Development in a Xenograft Model

**Figure 6:**
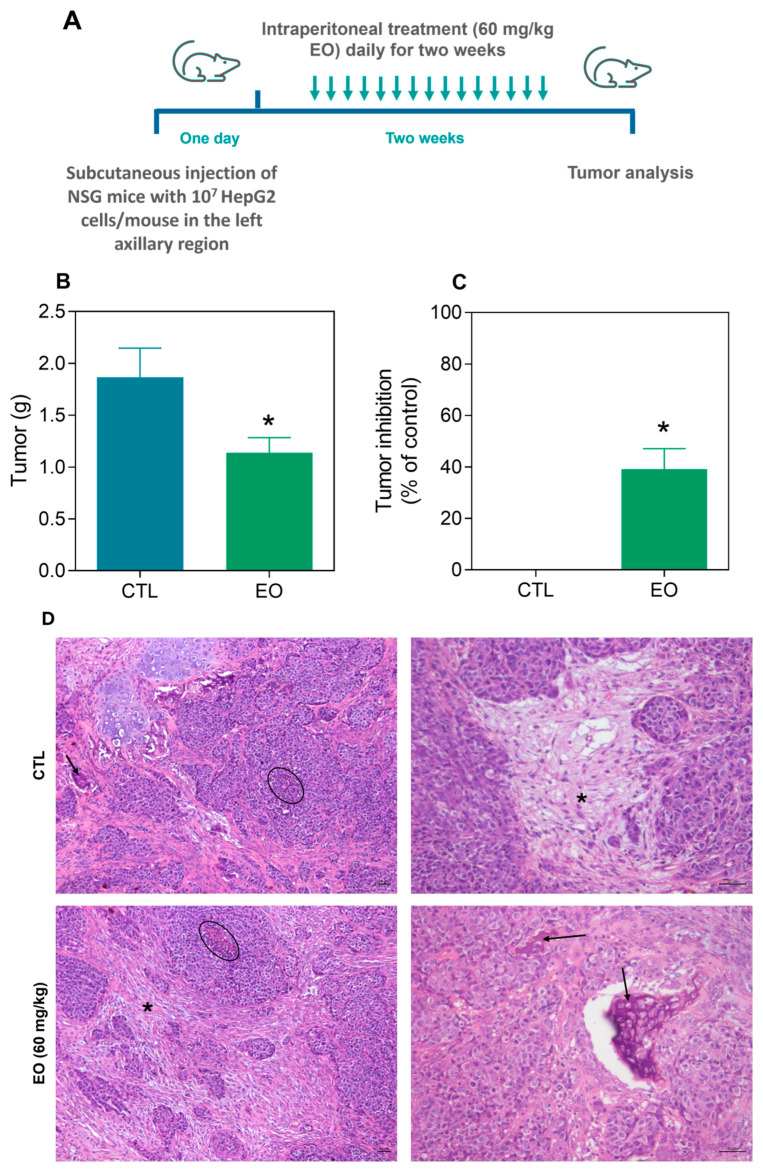
Antitumor effects of *A. amazonica* leaf EO in NSG mice xenografted with HepG2 cells. (**A**) Liver tumor xenograft model design. (**B**) Tumor weight and (**C**) tumor growth inhibition in animals treated intraperitoneally with EO (60 mg/kg) for two weeks. (**D**) Representative histological images of HepG2 tumor tissues. Arrows highlight regions of dystrophic calcification, necrotic areas are demarcated with circles, and fibrotic scar tissue is indicated by an asterisk. The mice that received 5% DMSO served as the vehicle control (CTL). The data are presented as the means ± SEMs for groups of 10 animals. * *p* < 0.05 compared with CTL, as assessed by two-tailed unpaired Student’s *t* test.

The in vivo efficacy of the EO from *A. amazonica* leaves on liver cancer development was evaluated in a xenograft model. NOD.Cg-*Prkdc^scid^Il2rg^tm1Wj^l/*SzJ (NSG) mice xenografted with HepG2 cells were treated with 60 mg/kg EO for two weeks (Figure 6A). After treatment, the mean tumor weight was 1.9 ± 0.3 g, while the EO-treated animals had a mean weight of 1.1 ± 0.2 (Figure 6B), indicating significant tumor inhibition of 39.2% (Figure 6C).

Histological analysis revealed that the morphological characteristics of the tumors were consistent with those of moderately differentiated hepatocellular carcinoma. The extracellular intratumoral matrix was predominantly composed of collagen fibers and exhibited limited vascularization. In some areas, neoplastic cells were arranged in island-like clusters embedded within a collagen-rich stroma. Extensive regions of coagulative necrosis were observed throughout the tumor mass and were frequently associated with the peripheral infiltration of inflammatory cells. Areas of granulation tissue and focal dystrophic calcification were also identified. Moreover, evidence of tumor invasion into adjacent muscle, adipose, and cartilaginous tissues was observed (Figure 6D).

Systemic toxicology evaluation was also performed in EO-treated NGS mice xenografted with HepG2 cells. All groups presented 100% survival rates, and no significant alterations in body or organ weights (liver, kidney, lung, or heart) were detected in any group (Figure S8).

The systemic toxicity of EO was evaluated through histological analysis of the heart, liver, lungs, and kidneys. The tissue architecture of the heart and kidneys remained preserved in both groups, with only mild vascular hyperemia observed as the sole alteration. In contrast, the hepatic parenchyma was partially preserved, with mild hydropic degeneration of hepatocytes. Vascular hyperemia was identified in the central venules and vessels of the portal triad, accompanied by infiltration of inflammatory cells, predominantly mononuclear, in these regions as well as in the perisinusoidal space. The pulmonary parenchyma exhibited marked structural alterations, including areas of atelectasis secondary to alveolar septal thickening caused by pneumocyte hyperplasia and hypertrophy. Moderate hyperemia was also observed in the pulmonary capillaries, along with inflammatory infiltrates predominantly composed of alveolar macrophages. Additionally, focal areas of necrosis were identified in the bronchial epithelium, along with foci of hemorrhage in the lung tissue (Figure S9).

In previous studies, *A. vepretorum* leaf EO complexed with β-cyclodextrin reduced the development of B16-F10 melanoma cells, and no signs of systemic toxicity were observed [25]. Similarly, *A. leptopetala* EO demonstrated antitumor activity in a 180 sarcoma tumor model, with no genotoxicity and only moderate gastrointestinal toxicity [23]. EO obtained from *A. neoinsignis* leaves also reduced HepG2 cell growth in a xenograft model [24]. The major chemical constituent of *A. amazonica* leaf EO, (*E*)-caryophyllene, also significantly reduces the tumor development of HCT116 cells in xenograft nude mice [34]. (*E*)-Caryophyllene also inhibited tumor growth and metastasis in a B16-F10 melanoma mouse model [40].

## 3. Conclusions

In summary, this study demonstrates, for the first time, the antitumor potential of *A. amazonica* leaf EO against liver cancer both in vitro and in vivo. The chemical composition of the EO revealed a predominance of bioactive compounds such as (*E*)-caryophyllene, 1,8-cineole, α-copaene, α-humulene, and α-pinene, which may contribute to its cytotoxic activity. EO treatment induced apoptosis in HepG2 cells through DNA fragmentation and loss of ΔΨm. In a xenograft model, EO reduced tumor development by 39.2%. These findings demonstrate that *A. amazonica* EO has promising properties as a natural therapeutic agent against liver cancer and justify further studies to characterize its pharmacological action and elucidate its molecular mechanisms of action, as well as its combination with clinically approved chemotherapeutics.

## 4. Materials and Methods

### 4.1. Botanical Material

Leaves of *A. amazonica* were collected in December 2021 at the Adolpho Ducke Forest Reserve, which is located near Manaus, Amazonas, Brazil (coordinates: 2°54′49.9” S, 59°58′43.6” W). The species was taxonomically identified by Professor Antônio Carlos Webber of the Federal University of Amazonas (UFAM). A voucher specimen was deposited in the Herbarium of the Department of Biology at UFAM (HUAM) under registration number #012098. The genetic material was registered in Brazil’s National System for the Management of Genetic Heritage and Associated Traditional Knowledge (SISGEN) under the registration code #A70EDCD.

### 4.2. EO Extraction

*A. amazonica* leaves were dried in a forced-air oven at 40 °C for 24 h and subsequently ground via a four-blade mill. EO extraction was performed by hydrodistillation via a Clevenger-type apparatus connected to a 4 L round-bottom flask. The mixture was heated to 100 °C, and extractions were conducted in triplicate, each with 300 g of plant material and sufficient distilled water to fill half the volume of the flask. The hydrodistillation process lasted for 3 h. The resulting EO was collected and dehydrated with anhydrous sodium sulfate to remove residual moisture and then stored in amber glass bottles under refrigeration to prevent chemical degradation. The EO yield was calculated as a percentage via the following formula: EO yield (%) = (mass of EO/mass of plant material) × 100.

### 4.3. Chemical Analysis of the EO

The chemical composition of the EO was analyzed via a TRACE GC ULTRA/ISQ gas chromatograph–mass spectrometer (Thermo Scientific) equipped with a TriPlus RSH autosampler operating in both gas chromatography–mass spectrometry and gas chromatography–flame ionization detection modes. Separation was achieved via the use of a DB-5MS fused silica capillary column (30 m × 0.25 mm × 0.25 μm) coated with 5% phenylarylene and 95% dimethylpolysiloxane. Helium was employed as the carrier gas at a constant flow rate of 1.0 mL/min. The oven temperature program was as follows: initial temperature of 40 °C (held for 4 min), increased at 4 °C/min to 240 °C, ramped at 10 °C/min to 280 °C, and held for 2 min. The injector temperature was set at 250 °C, and the detector temperature was 280 °C, following the method described by Silva et al. [41].

The samples were prepared by dissolving 10 mg of EO in 1 mL of dichloromethane to obtain pesticide residue. A 1 μL aliquot was injected in split mode (1:25 ratio). The retention indices were calculated via a homologous series of *n*-alkanes (C_8_–C_20_) and the equation proposed by van den Dool and Kratz [17]. Chromatographic data, including retention times and peak areas, were acquired and processed electronically. Mass spectra were recorded at 70 eV with a scan interval of 0.5 s and a mass range of 40–550 Da. The same column and stationary phase used for GC–FID were applied in the GC–MS analysis.

The EO constituents were identified by comparing the obtained mass spectra and calculated retention indices with those reported in the literature [18]. The relative abundance of each compound was expressed as a percentage of the total chromatographic peak area.

### 4.4. Cells

This study used a panel of cancer cell lines that included HCT116 (human colorectal cancer, ATCC code #CCL-247), HepG2 (human liver cancer, ATCC code #HB-8065), MDA-MB-231 (human breast cancer, ATCC code #HTB-26), MCF-7 (human breast cancer, ATCC code #HTB-22), 4T1 (mouse breast cancer, ATCC code #CRL-2539), and B16-F10 (mouse melanoma, ATCC code #CRL-6475) cells, along with the noncancerous human lung fibroblast line MRC-5 (ATCC code #CCL-171). All cell lines were obtained from the American Type Culture Collection (ATCC; Manassas, VA, USA) and cultured according to standard ATCC protocols for animal cell maintenance [42], and only mycoplasma-free cells were used.

### 4.5. Alamar Blue Assay

Cell viability was evaluated via the alamar blue assay, following the protocol described by Ahmed et al. [43]. The cells were seeded into 96-well plates and treated with the EO, which had been previously dissolved in dimethyl sulfoxide (DMSO; Vetec Química Fina Ltd.a., Duque de Caxias, RJ, Brazil). Eight different EO concentrations were tested, and the cells were incubated for 72 h. Doxorubicin hydrochloride (IMA S.A.I.C., Buenos Aires, Argentina) was used as a positive control. After the incubation period, 20 μL of a 1 mM resazurin solution (Sigma–Aldrich, St. Louis, MO, USA) was added to each well. The absorbance readings were taken at 570 and 600 nm via a SpectraMax 190 microplate reader (Molecular Devices, Sunnyvale, CA, USA). IC_50_ values and corresponding 95% confidence intervals (95% CIs) were calculated via nonlinear regression analysis.

### 4.6. Flow Cytometry Assays

The quantification of apoptotic cells was performed via the use of the fluorescent dyes YO-PRO-1 (Sigma–Aldrich Co.) and PI (BD Biosciences, San Jose, CA, USA), adapted from Idziorek et al. [32]. The cells were stained with a solution containing 0.1 µM YO-PRO-1 and 1.5 µM PI, and fluorescence was measured via flow cytometry.

The ΔΨm was assessed via rhodamine-123 staining, as described by Sureda et al. [44]. The cells were incubated with 1 µg/mL rhodamine-123 (Sigma–Aldrich Co.) for 15 min at 37 °C in the dark. After washing, the fluorescence intensity was analyzed via flow cytometry.

To assess internucleosomal DNA fragmentation and cell cycle distribution, the cells were stained with PI following the methods of Nicoletti et al. [45]. Briefly, the cells were suspended in permeabilization buffer containing 0.1% Triton X-100, 2 μg/mL PI, 0.1% sodium citrate, and 100 μg/mL RNase (all from Sigma–Aldrich Co.), followed by fluorescence analysis via flow cytometry.

All flow cytometric analyses were performed via a BD LSRFortessa cytometer, with data acquisition and analysis performed via BD FACSDiva (BD Biosciences) and FlowJo 10 (FlowJo LLC; Ashland, OR, USA). For each sample, at least 10^4^ events were recorded. Cellular debris was excluded from the analysis, and individual cell populations were gated based on forward scatter height vs. area (FSC-H vs. FSC-A) and/or side scatter height vs. area (SSC-H vs. SSC-A) parameters.

### 4.7. Animals

Twenty NSG mice (specific pathogen-free, male and female, 8–10-week-old, 20–25 g) were obtained and housed in the animal facility of Instituto Gonçalo Moniz—FIOCRUZ (Salvador, Bahia, Brazil). The animals were kept under control in polyacrylic cages (with a maximum of five animals per cage), with ad libitum access to food and water. A 12 h light/dark cycle was applied (lights on at 7:00 AM). All procedures were approved by the Institutional Animal Care and Use Committee of the Instituto Gonçalo Moniz—FIOCRUZ (protocol number 01/2021), which are in accordance with international regulations.

### 4.8. Human Liver Tumor Xenograft Model

To establish the human liver tumor xenograft model, HepG2 cells (10^7^ cells in 500 µL per animal) were injected subcutaneously into the left axillary region. Twenty-four hours after inoculation, the mice received daily intraperitoneal treatments (200 µL per animal) for two weeks. The animals were randomly divided into two groups: (1) the control group receiving vehicle (5% DMSO; n = 10) and (2) the treatment group receiving EO at a dose of 60 mg/kg (n = 10). At the end of the treatment period, the animals were euthanized by an anesthetic overdose (ketamine-xylazine), and the tumors were excised and weighed. Tumor growth inhibition was calculated via the following formula: inhibition ratio (%) = [(A − B)/A] × 100, where A is the mean tumor weight of the control group and B is the mean tumor weight of the treated group.

Toxicological evaluations included monitoring body weight at the beginning and end of the experiment. The animals were also observed daily for clinical signs of toxicity. Major organs (liver, kidneys, lungs, and heart) were removed, weighed, and examined macroscopically for signs of pathological changes such as lesions, discoloration, or hemorrhage. Histopathological analysis of tumors and organs was conducted via light microscopy after fixation in 4% formaldehyde. The tissue sections were stained with hematoxylin and eosin (H&E) for gross morphology and with periodic acid-Schiff (PAS) for evaluation of the liver and kidney. Microscopic examinations were conducted at magnifications of ×4, ×100, ×200, and ×400. Histological findings were scored semiquantitatively as negative (0), mild (+1), moderate (+2), or severe (+3). Representative images were acquired via a Leica LMD6500 microscope with LAS v4 image acquisition software.

### 4.9. Statistical Analysis

The data are expressed as the means ± standard errors of the means (SEMs) or as IC_50_ values accompanied by 95% CIs and are based on a minimum of three independent experiments (biological replicates) conducted in duplicate or triplicate (technical replicates). Statistical analysis between groups was performed via two-tailed unpaired Student’s *t* test or one-way analysis of variance (ANOVA) followed by Dunnett’s post hoc test. Differences were considered statistically significant at *p* < 0.05. All the statistical analyses were carried out via GraphPad Prism software version 8 (GraphPad Software, San Diego, CA, USA).

## Figures and Tables

**Table 1:** Chemical composition of the EO obtained from the leaves of *A. amazonica*.

Compounds		AI ^a^	AI ^b^	Peak Area (%)
1	α-pinene	929	932	5.13 ± 1.72
2	α-fenchene	943	945	0.32 ± 0.20
3	sabinene	969	969	0.11 ± 0.02
4	β-pinene	971	974	1.72 ± 0.38
5	myrcene	990	988	0.62 ± 0.24
6	α-terpinene	1013	1014	0.12 ± 0.03
7	*O*-cymene	1021	1022	0.18 ± 0.02
8	1,8-cineole	1027	1026	13.93 ± 0.84
9	(*Z)*-β-ocimene	1038	1032	0.11 ± 0.05
10	(*E*)-β-ocimene	1048	1044	0.21 ± 0.08
11	γ-terpinene	1056	1054	0.15 ± 0.02
12	terpinolene	1085	1086	1.04 ± 0.42
13	linalool	1099	1095	2.91 ± 0.79
14	terpinen-4-ol	1174	1174	0.23 ± 0.09
15	α-terpineol	1188	1186	2.16 ± 0.76
16	geraniol	1254	1249	0.73 ± 0.41
17	δ-elemene	1336	1335	0.19 ± 0.08
18	α-cubebene	1349	1348	0.32 ± 0.04
19	cyclosativene	1365	1369	0.31 ± 0.04
20	α-copaene	1375	1374	7.77 ± 0.36
21	β-elemene	1391	1389	0.60 ± 0.36
22	(*E*)-caryophyllene	1420	1417	32.01 ± 3.98
23	β-copaene	1427	1430	1.10 ± 0.18
24	γ-elemene	1433	1434	0.15 ± 0.01
25	*trans*-α-bergamotene	1435	1432	0.40 ± 0.19
26	aromadendrene	1437	1439	0.56 ± 0.03
27	6,9-guaiadiene	1442	1442	0.24 ± 0.10
28	α-humulene	1452	1452	7.15 ± 1.10
29	*allo*-aromadendrene	1459	1458	0.12 ± 0.02
30	γ-muurolene	1475	1478	0.76 ± 0.07
31	germacrene D	1480	1480	1.86 ± 1.43
32	β-selinene	1484	1489	0.36 ± 0.03
33	*cis*-β-guaiene	1490	1492	0.12 ± 0.05
34	bicyclogermacrene	1495	1500	2.07 ± 0.35
35	α-muurolene	1499	1500	0.77 ± 0.05
36	γ-cadinene	1512	1513	0.29 ± 0.04
37	δ-cadinene	1522	1522	1.17 ± 0.05
38	α-calacorene	1541	1544	0.14 ± 0.03
39	elemol	1548	1548	0.88 ± 0.09
40	germacrene B	1555	1559	0.37 ± 0.08
41	caryophyllene oxide	1579	1582	0.52 ± 0.09
42	guaiol	1596	1600	1.40 ± 0.30
43	eremoligenol	1627	1629	0.43 ± 0.14
44	γ-eudesmol	1630	1630	1.54 ± 0.48
45	hinesol	1637	1640	0.12 ± 0.07
46	cubenol	1640	1645	0.17 ± 0.11
47	β-eudesmol	1648	1649	1.76 ± 0.62
48	α-eudesmol	1651	1652	2.59 ± 0.90
49	bulnesol	1665	1670	0.26 ± 0.09
Monoterpenes				29.67
Sesquiterpenes				68.50
Total not identified				1.83
Total identified				98.17

Note: The data are presented as the means ± S.D. of two analyses. RI (retention indices): This index was calculated on a TR-5MS capillary column (30 m × 0.25 mm × 0.25 µm) according to Van Den Dool and Kratz [17], which is based on a homologous series of normal alkanes, according to Adams [18].

**Table 2:** In vitro cytotoxic effects of *A. amazonica* leaf EO.

Cells	Histological Type	IC_50_ and 95% CIs (in μg/mL)	
		DOX	EO
Cancer cells			
HCT116	Human colon cancer	0.20 0.11–0.35	21.42 17.60–26.07
HepG2	Human liver cancer	0.04 0.03–0.05	14.72 7.54–28.75
MDA-MB-231	Human breast cancer	0.62 0.37–1.02	16.20 6.59–29.85
MCF-7	Human breast cancer	0.36 0.20–0.63	44.07 39.37–48.52
4T1	Mouse breast cancer	0.60 0.31–0.96	21.65 16.41–29.90
B16-F10	Mouse melanoma	0.06 0.03–0.09	32.16 27.46–38.34
Noncancerous cells			
MRC-5	Human lung fibroblast	0.43 0.18–1.03	39.41 30.88–50.29

Note: Doxorubicin (DOX) was used as a positive control.

## Data Availability

The data presented in this study are available in the Supplementary Materials.

## Supplementary Materials

The following supporting information can be downloaded at: https://www.mdpi.com/article/10.3390/molecules30153248/s1, Figure S1: Illustration of the experimental workflow; Figure S2: Chromatogram of the total ions; Figure S3: Mass spectrum of α-pinene; Figure S4: Mass spectrum of 1,8-cineole; Figure S5: Mass spectrum of α-copaene; Figure S6: Mass spectrum of (*E*)-caryophyllene; Figure S7: Mass spectrum of α-humulene; Figure S8: The body weight and relative organ weight; Figure S9: Representative photomicrographs of organs.
