# Supplementary material for: In Vitro and In Vivo Efficacy of the Essential Oil from the Leaves of Annona amazonica R.E. Fries (Annonaceae) Against Liver Cancer
Source: Molecules. 2025 Aug 2;30(15):3248. doi: 10.3390/molecules30153248 (PMC12348787; doi:10.3390/molecules30153248)
Supplement: Supplementary file 1 [file molecules-30-03248-s001.zip › molecules-3748800-supplementary.pdf]

Supplementary Material

In vitro and in vivo efficacy of the essential oil from the leaves of *Annona amazonica* R.E. Fries (Annonaceae) against liver cancer

Maria V. L. de Castro[1,†], Milena C. F. de Lima[2,†], Gabriela A. da C. Barbosa[1], Sabrine G. Carvalho[1], Amanda M. R. M. Coelho[1], Luciano de S. Santos[1], Valdenizia R. Silva[1], Rosane B. Dias[1,3], Milena B. P. Soares[1,4], Emmanoel V. Costa[2,5]*, Daniel P. Bezerra[1,*]

[1]Gonçalo Moniz Institute, Oswaldo Cruz Foundation (IGM-FIOCRUZ/BA), Salvador, Bahia, 40296-710, Brazil.

[2]Department of Chemistry, Institute of Exact Sciences, Federal University of Amazonas (UFAM), Manaus, Amazonas, 69080-900, Brazil.

[3]Department of Biological Sciences, State University of Feira de Santana, Feira de Santana, Bahia, 44036-900, Brazil.

[4]SENAI Institute for Innovation in Advanced Health Systems, SENAI CIMATEC, Salvador, BA 41650-010, Brazil.

[5]Postgraduate Program in Chemistry, Institute of Exact Sciences, Federal University of Amazonas (UFAM), Manaus, Amazonas, 69080-900, Brazil.

† These authors contributed equally to this work.

*Corresponding authors:

E. V. Costa (e-mail: evc@ufam.edu.br); D. P. Bezerra (e-mail: daniel.bezerra@fiocruz.br); Tel./Fax: +55-92-3305-1181 Ramal 2870 (E.V. Costa); Tel./Fax: +55-71-3176-2272 (D. P. Bezerra).

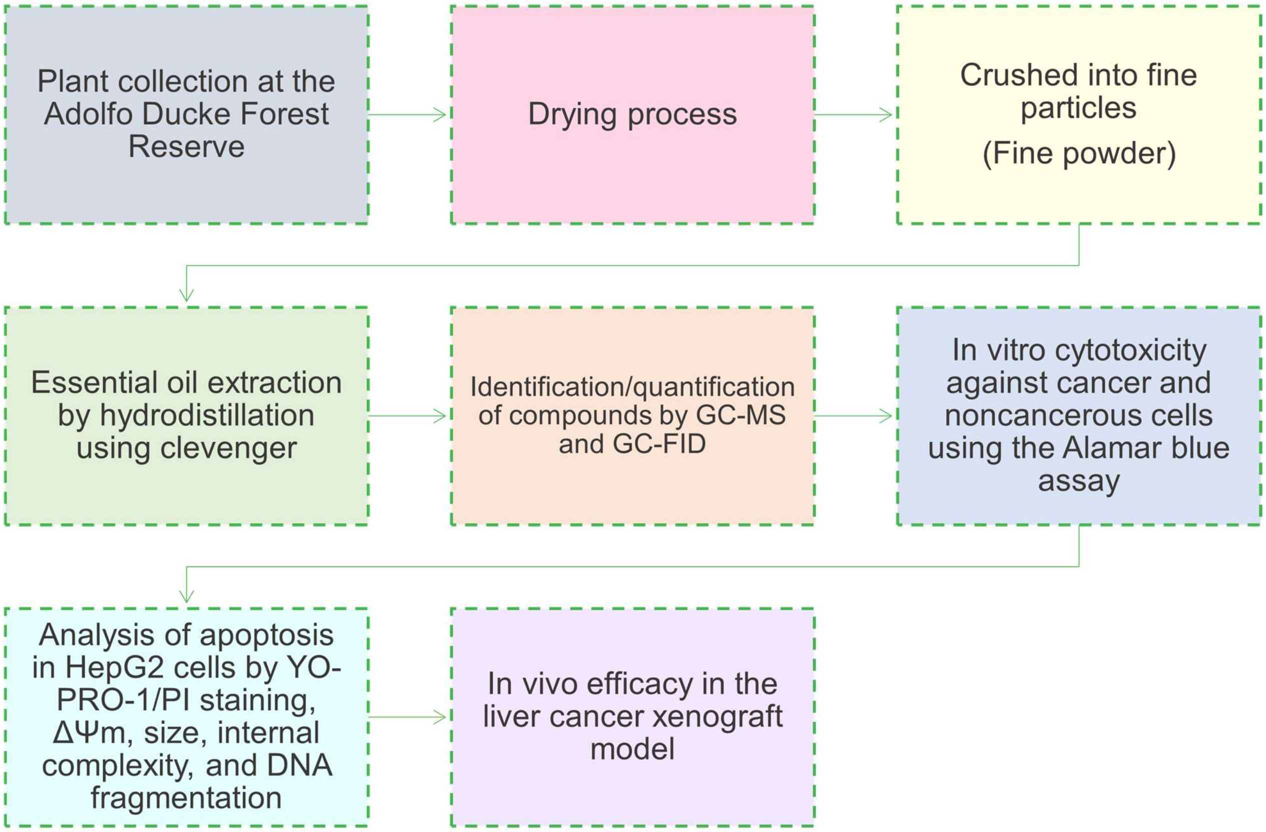

Figure S1. Illustration of the experimental workflow performed.

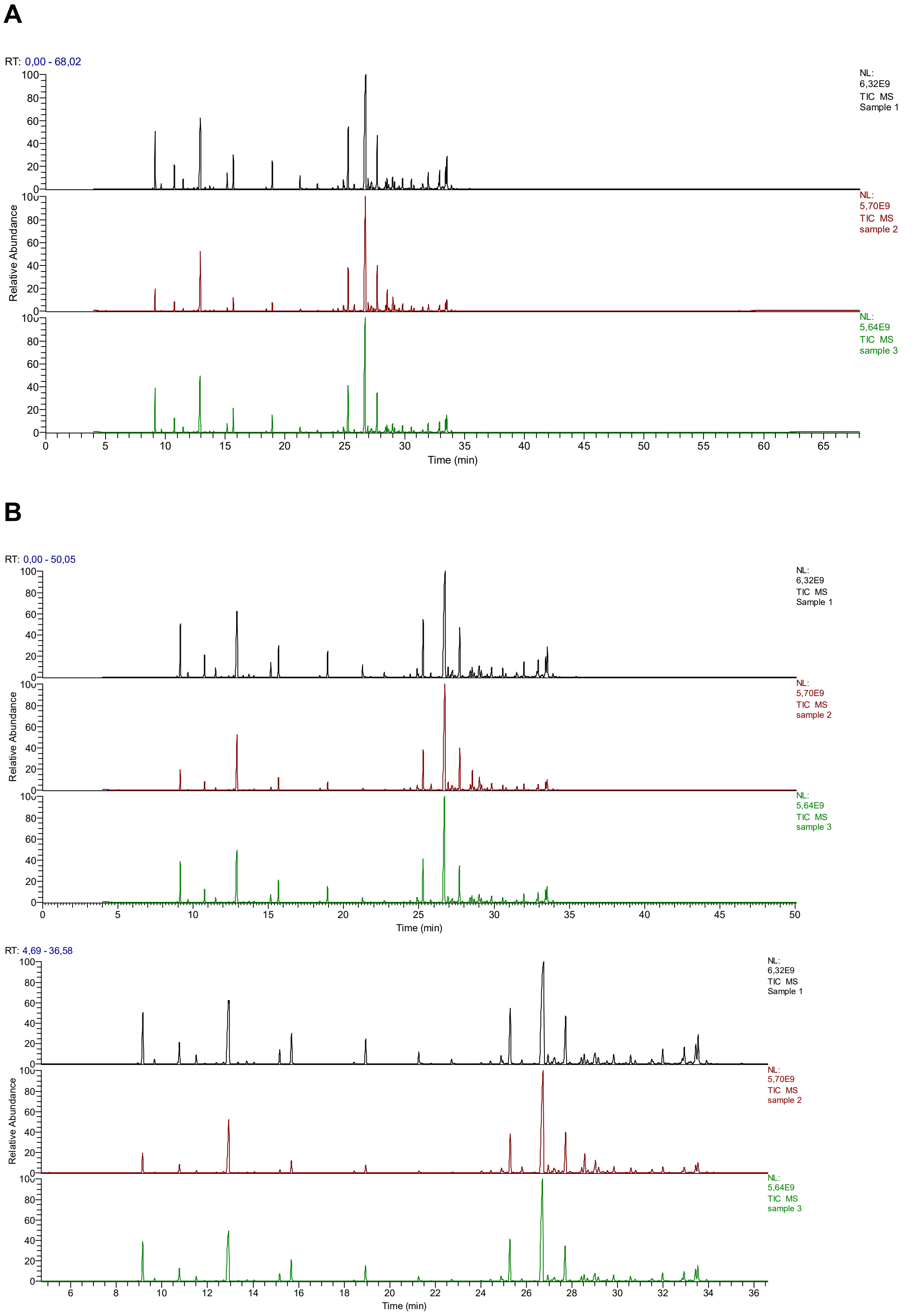

Figure S2. (**A**) Chromatogram of the total ions of *A. amazonica* leaf EO (triplicate); (**B**) Enlargement of the region between 6 min and 36 min.

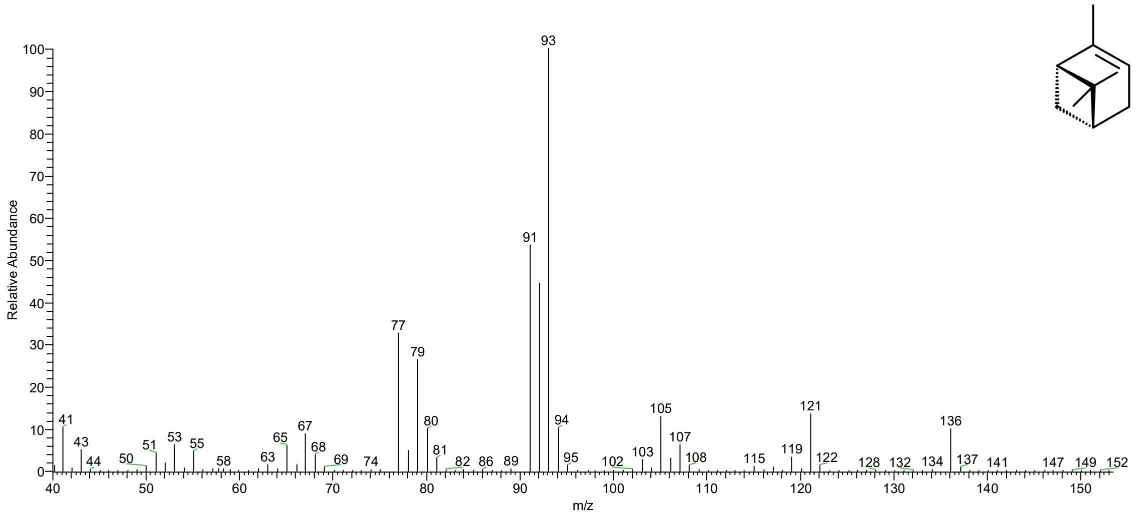

Figure S3. Mass spectrum of α-pinene.

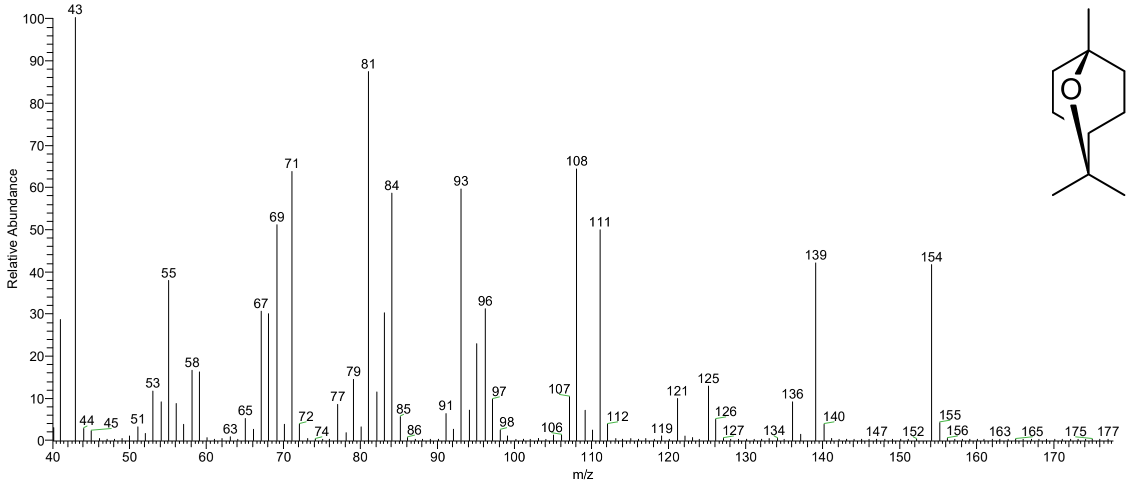

Figure S4. Mass spectrum of 1,8-cineole.

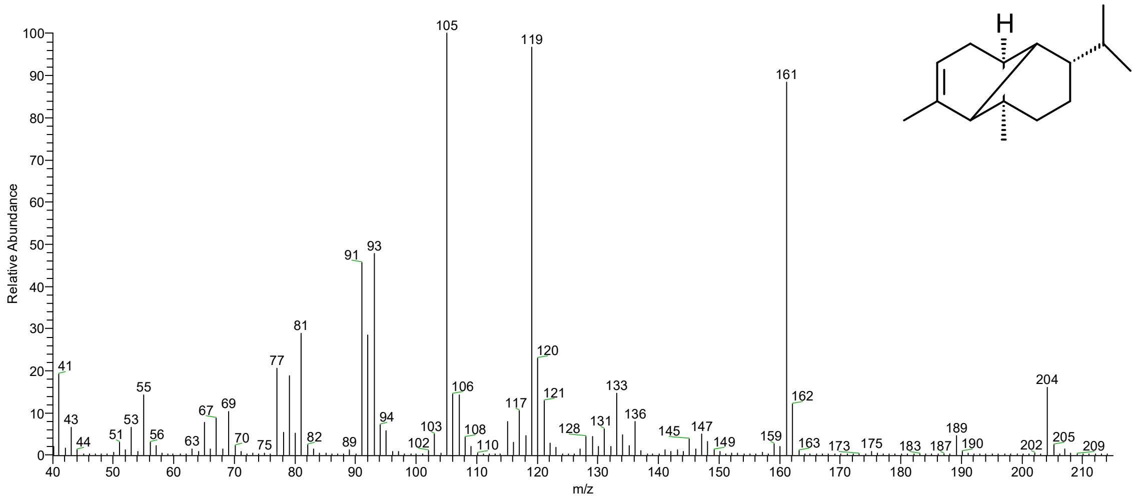

Figure S5. Mass spectrum of α-copaene.

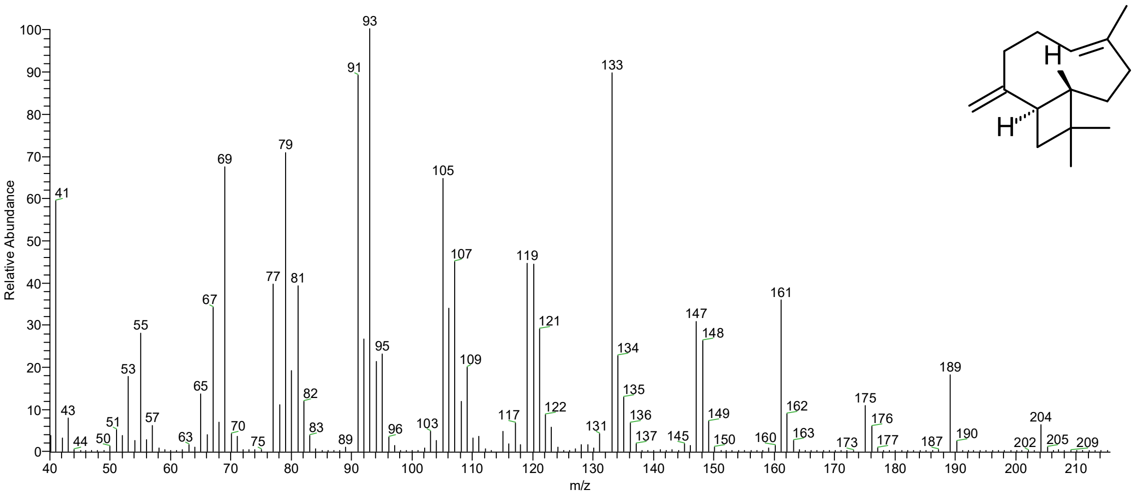

Figure S6. Mass spectrum of (*E*)-caryophyllene.

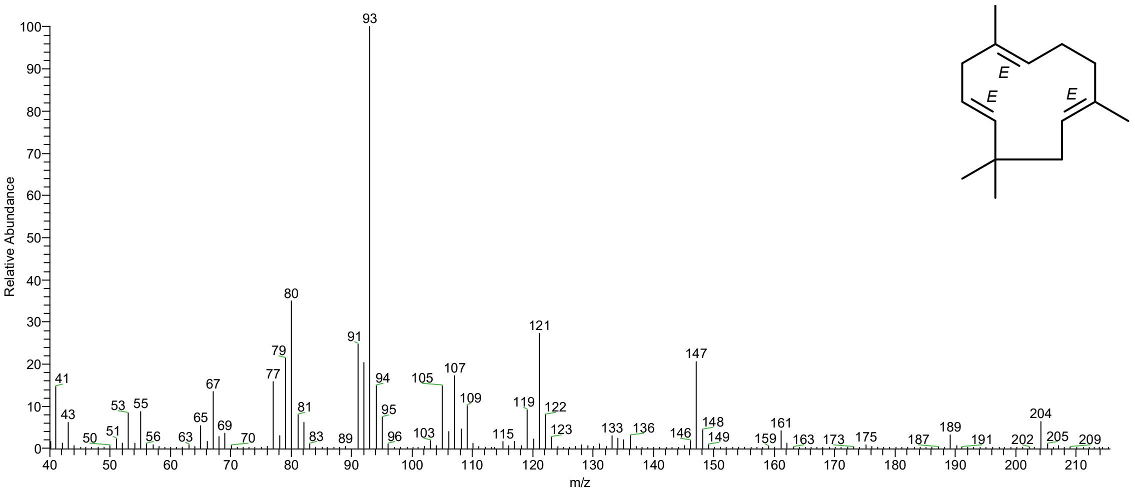

Figure S7. Mass spectrum of α-humulene.

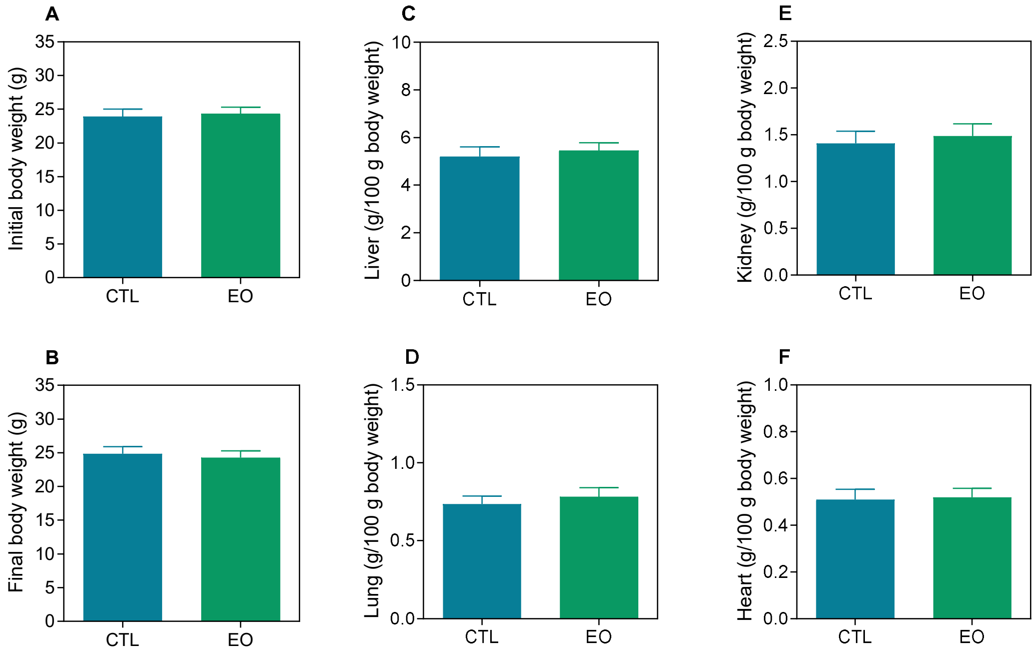

Figure S8. Evaluation of systemic toxicity after treatment with EO of *A. amazonica* leaf in NSG mice xenografted with HepG2 cells. (**A**, **B**) Monitoring of body weight before and after treatment. (**C**–**F**) Relative weights of liver, kidneys, lungs, and heart. Mice received daily intraperitoneal injections of EO (60 mg/kg) for two weeks. The vehicle control group (CTL) received 5% DMSO. Data are expressed as the mean ± SEM of 10 animals per group.

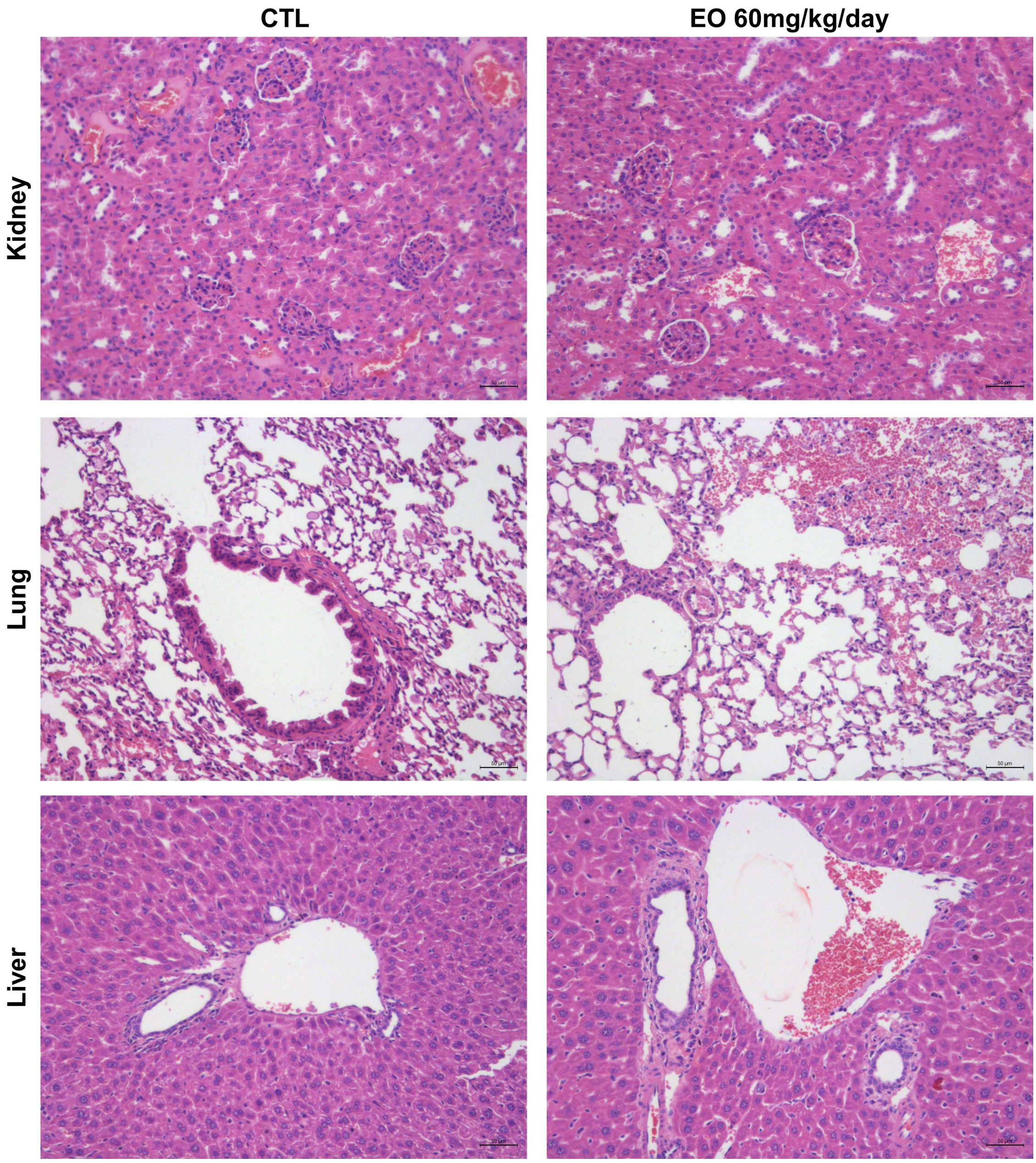

Figure S9. Representative photomicrographs of organs. The treatments (60 mg/kg EO) were injected intraperitoneally into the mice daily for two weeks. The vehicle (5% DMSO) was used as a negative control (CTL).
